# Effects of bone cement on intervertebral disc degeneration

**DOI:** 10.3892/etm.2014.1531

**Published:** 2014-02-10

**Authors:** HUI ZHAO, CAI-FANG NI, JIAN HUANG, SU-MING ZHAO, WEI-WEI GU, HAO JIANG, LONG CHEN, TIAN-SI TAN

**Affiliations:** 1 Department of Interventional Radiology, The Hospital Affiliated of Nantong University, Nantong, Jiangsu 226001, P.R. China; 2 Department of Interventional Radiology, The First Affiliated Hospital of Soochow University, Nantong, Jiangsu 215006, P.R. China; 3 Department of Orthopedics, The First Affiliated Hospital of Soochow University, Nantong, Jiangsu 215006, P.R. China

**Keywords:** vertebroplasty, polymethyl methacrylate, calcium phosphate cement, intervertebral disc, degeneration, magnetic resonance imaging

## Abstract

Percutaneous vertebroplasty (PVP) is popular for the treatment of intractable pain due to vertebral collapse from various lesions, intervertebral disk leakage of cement is a frequent complication. The aim of this study was to determine whether bone cement causes disc degeneration, and to evaluate the degree of intervertebral disc degeneration (IDD) according to the time period following cement injection, and the type and volume of cement injected. Sixteen dogs were randomly divided into two groups that were sacrificed at 12 or 24 weeks following cement injection. Five intervertebral discs in each dog were studied, including one control untreated disc and four discs randomly injected with polymethylmethacrylate (PMMA) or calcium phosphate cement (CPC) in two quantities. Radiographic and magnetic resonance imaging (MRI) studies were performed prior to animal sacrifice. T2-weighted mid-sagittal images of the discs were qualitatively analyzed for evidence of degeneration by calculating the MRI index, and all harvested discs were studied histopathologically. IDD was not evident in control discs. Univariate analysis revealed significant differences in the MRI index and the histological grade of disc degeneration in terms of the time period following cement injection, as well as the type and volume of cement injected. Result indicate that direct contact with PMMA and CPC can lead to IDD. However, IDD induced by PMMA was more severe than that induced by CPC. The extent of IDD was found to correlate with the time period post-cement injection and the volume of cement injected into the disc. PMMA and CPC may lead to intervertebral disc degeneration. Intervertebral disc degeneration induced by PMMA is more serious than that of CPC. The degree of intervertebral disc degeneration is correlative to the time after operation and the doses of bone cement.

## Introduction

Percutaneous vertebroplasty (PVP) is a minimally invasive procedure that involves radiographically guided injection of bone cement directly into the vertebral body (1). Polymethylmethacrylate (PMMA) and calcium phosphate cement (CPC) are common types of bone cement used for PVP. In 1987, Galibert *et al* (2) described PVP as a treatment for painful, aggressive, vertebral hemangioma. Although PVP is a minimally invasive and safe procedure, it is associated with a high complication rate (3). Cement leakages account for the majority of symptomatic complications (1,3,4). While intradiscal cement leakage is generally asymptomatic, it may have late mechanical consequences on the adjacent mobile spinal segments (5,6). Intradiscal cement leakage is commonly observed following PVP. Mirovsky *et al* (1) reported intradiscal cement leakage following PVP in 41% of patients. The age and gender of patients, the type of fracture, the volume of injected PMMA, and the liquid consistency of PMMA were not found to be statistically significant risk factors for intradiscal cement leakage. In a study by Pitton *et al* (7), intradiscal cement leakage was detected in 27.5% of patients who had undergone PVP, and routes of leakage included fractured endplates, vacuum clefts or iatrogenic perforation of the endplate by the needle tip. Although leakage is largely asymptomatic, it may have long-term effects on the disc and adjacent vertebrae. Recently published data indicate that large-disc cement leaks may predispose individuals to the collapse of adjacent vertebrae (8). The association between intradiscal cement leakage and the occurrence of new adjacent osteoporotic vertebral compression fractures (OVCF) was substantiated by the identification of the following volumetric correlation: A higher intradiscal leakage volume is associated with an increased likelihood of new adjacent OVCF. This volumetric effect may explain the diverse findings across studies, i.e., depending on the mean leakage volume, intradiscal cement leakage may or may not be a risk factor for new fractures. Therefore, intradiscal cement leakage, particularly large volumes (≥1 ml), should be avoided (8). Although rates of complications for PVP are low, cement leakage occurs in up to 90% of patients. Current evidence indicates that sequelae of cement leakage may be more common and clinically relevant than previously considered (9).

The present study aimed to investigate whether bone cement can lead to intervertebral disc degeneration (IDD), and evaluate the degree of IDD relative to the type and volume of cement injected into the disc space.

## Materials and methods

### Experimental animals

Adult dogs (n=16; each ~16 kg) were supplied by the Experimental Animal Center of Soochow University (Nantong, China). The animal use protocol was approved by the Institutional Animal Care and Use Committee of Soochow University (approval number SVXK; SU2002-0037). This study was performed in accordance with the Guide for the Care and Use of Laboratory Animals published by the National Institutes of Health (1996). The dogs were randomly divided into two groups according to sacrifice time (12 or 24 weeks post-cement injection). In each dog, five lumbar intervertebral discs (L1–L2 to L5–L6) were studied. One disc was untreated and acted as the control group, whereas the other four discs were injected with PMMA (Corin Ltd., Cirencester, UK) or CPC (Shanghai Rebone Biomaterials Co., Ltd, Shanghai, China). A total of 0.1 or 0.3 ml cement was injected. The dogs were randomized into five groups: A, control; B1, 0.1 ml PMMA; B2, 0.3 ml PMMA; C1, 0.1 ml CPC; and C2, 0.3 ml CPC (Table I).

### Experimental procedure

**Figure 1:**
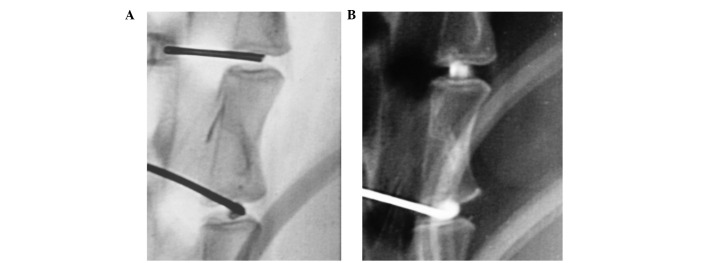
Lateral view of the X-ray film prior to and following cement injection.

The dogs were anesthetized with 3% pentobarbital sodium (1 mg/kg i.v.) and hair was shaved from the left flank and mid-back. Next, the animals were positioned in left lateral recumbence on the operating table, and the skin of the spine was sterilized and then fixed. Under the guidance of fluoroscopy from a digital subtraction angiography machine (Agniostar Plus; Siemens AG, Munich, Germany), an 18G spinal needle (Terumo Corporation, Tokyo, Japan) was inserted into the lumbar intervertebral disc of the dogs. The magnification function was used to ensure that a one-shot puncture was achieved. The PMMA was mixed with barium sulfate, resulting in a 20% ratio of PMMA powder and liquid. The cement mixture was prepared once the spinal needle was positioned, with the tip of the needle reaching the center of the disc. The cement is ready for injection when it drips slowly like melted ice cream from the tip of the 1 ml syringe. The needle was removed after the chosen cement was injected. Lateral plane radiographs of the L1–L6 intervertebral discs were obtained prior to and following injection (Fig. 1). The collimator-to-film distance was 80 cm, with an exposure of 120 mAs and penetration power of 54 kVp.

### Magnetic resonance imaging (MRI) examination and analysis

A 1.5-T clinical magnet was used for MRI scans. The anesthetized dogs were placed in a supine position within the magnet field, with the lumbar region centered over a 10-inch diameter circular surface coil (General Electric Healthcare, Cleveland, OH, USA). A coronal T2-weighted localizer image [repetition time (TR), 1,445 ms; echo time (TE), 37 ms] was used to establish the position of the lumbar discs from L1–L2 to L5–L6. A 4-mm thick mid-sagittal section (field of view, 12×9-cm; matrix size, 256×192 cm) was imaged by using a T2-weighted imaging sequence (TR, 4,188 ms; TE, 112 ms) to highlight the signal from the nucleus pulposus. T2-weighted axial images (TR, 3,500 ms; TE, 100 ms) and T1-weighted mid-sagittal images (TR, 324 ms; TE, 12 ms) were also obtained.

The T2-weighted mid-sagittal images of the discs were qualitatively analyzed for evidence of degeneration by calculating the MRI index using Sobajima’s method (10). The MRI outcome, also know as the MRI index (the product of the nucleus pulposus area and average signal intensity), was used as a measure of the degenerative changes in the nucleus pulposus. Quantitative analysis of these images was performed with a picture archiving and communications system in which the nucleus pulposus of each disc was outlined on the screen and the computer mouse was used to define the region of interest. The intra-observer reliability in classifying disc degeneration via MRI was assessed by having the same examiner re-evaluate all images >1 month after the initial assessment. To assess inter-observer reliability, two senior authors, who have >15 years of experience as board-certified surgeons, independently performed the rating. The degree of agreement beyond chance was determined according to Cohen’s κ statistics (11).

### Histology examination

The dogs were sacrificed by intravascular embolization of air. The L1–L6 spinal segments were removed, and following 48 h of fixation in 10% formalin, each segment was sawed open. The distance between the superior and inferior endplates was 1 cm. The intact intervertebral disc, and the superior and inferior endplates were placed into decalcifying fluid for 4 weeks. Once decalcification was complete, each segment was cut sagittally into four parts, in series through the center of the vertebral body. Each part had a thickness of 3 mm and was dehydrated for 12 h. Following paraffin embedding, each part was cut into six serial sections (5 μm each), yielding a total of 24 sections.

To observe the changes in the nucleus pulposus, annulus fibrosus and cartilage endplate, the specimens were stained with hematoxylin and eosin. IDD pathological scores were reviewed according to the standards used by Masuda *et al* (12).

A histological grading scale based on four categories of degenerative changes was used to assess the annulus fibrosus, the border between the annulus fibrosus and the nucleus pulposus, the cellularity of the nucleus pulposus, and the matrix of the nucleus pulposus, using mid-sagittal sections (Table I). The grades ranged between 4 and 12. A normal disc was scored as grade 4, scoring 1 point for each of the four categories listed above for a total of 4 points. Grade 12 was representative of severe degeneration, with 3 being the maximum number of points that could be obtained in each category.

### Statistical analysis

Data are presented as the mean ± standard error. Intra- and inter-group variability was accounted for by mixed model analysis. Statistical analysis was performed using SPSS 17.0 software (SPSS, Inc., Chicago, IL, USA).

## Results

### MRI findings

**Figure 2:**
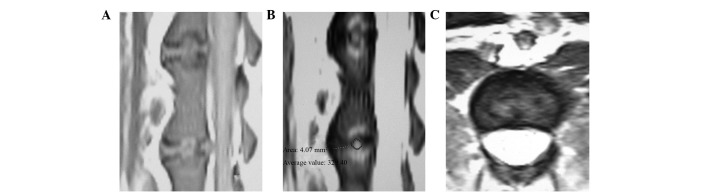
Images at 24 weeks after injection of 0.3 ml polymethylmethacrylate (A) T1-weighted image shows irregular and patchy low signals in the intervertebral space, where the intervertebral disc exhibited an abnormal shape. (B) T2-weighted images show that the area of relatively high signal declined. (C) Axial T2-weighted image shows the irregular shape of the nucleus pulposus, and the patchy high signals in the annulus fibrosus, which did not have a clear boundary with the nucleus pulposus.

**Figure 3:**
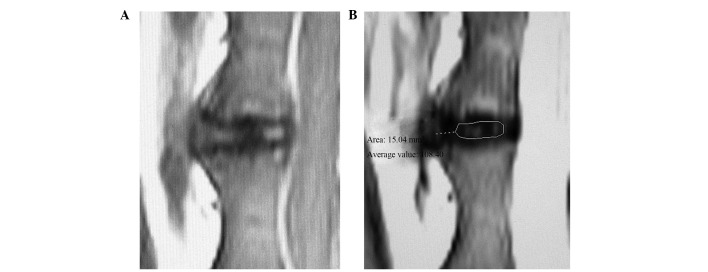
Images at 24 weeks after injecting 0.1 ml polymethylmethacrylate. (A) T1-weighted images reveal irregularity in the intervertebral space. (B) T2-weighted images demonstrate that the area of relatively high signal declined. Note the region of relative high signal where the degeneration of the nucleus pulposus happened was outlined on the screen and the computer mouse was used to define the region of interest.

The architectures of the annulus fibrosus and nucleus pulposus were intact in the control group at 12 and 24 weeks. The intervertebral space in the cement groups exhibited varying levels of stenosis at 12 and 24 weeks. The superior and inferior edges of the vertebral body at the corresponding intervertebral space were coarse. In the T2-weighted images, signals from the nucleus pulposus were not uniform and decreased at various rates. The center of the nucleus pulposus had a much lower signal relative to the control group, with an irregular and patchy appearance. The area of relatively high signal was diminished compared to control group and the shape of the nucleus pulposus was irregular (Fig. 2). A patchy streak of high signals was observed in the annulus fibrosus, which had a clear boundary with regard to the nucleus pulposus. Irregular and patchy low signals were observed in the intervertebral space in the T1-weighted images, and the intervertebral disc had an abnormal shape (Fig. 3). Figs. 2 and 3 demonstrate that specific vertebral bodies near the injected discs exhibited medullar edema, fat degeneration and Schmorl’s nodule formation.

**Figure 4:**
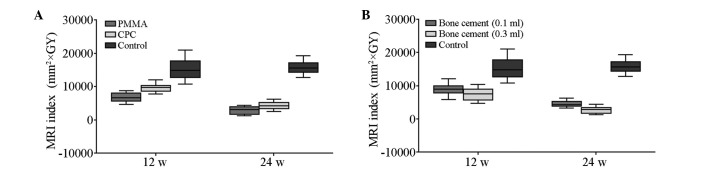
(A) MRI indexes for the PMMA and CPC subgroups in the 12- and 24-week groups. (B) MRI indexes for the various quantities of cement subgroups in the 12- and 24-week groups. MRI, magnetic resonance imaging; PMMA, polymethylmethacrylate; CPC, calcium phosphate cement.

With regard to the MRI index, no significant difference was observed at 12 and 24 weeks for cement and control groups (t=0.505; P>0.05; Table II). A significant difference in the MRI index of all cement subgroups was observed at 12 and 24 weeks compared with the control group. A significant difference was also observed between the PMMA groups and the CPC groups at 12 and 24 weeks (Fig. 4).

### Pathology findings

At 12 and 24 weeks, the control group had a normal boundary between the annulus fibrosus and the nucleus pulposus, and calcification in the cartilage endplate was not observed. The vascular bed was not reduced, and the cells of the nucleus pulposus were normal. The interstitial substance of the nucleus pulposus was not condensed. Although ruptures were present in certain samples, disruption was absent in the annulus fibrosus.

**Figure 5:**
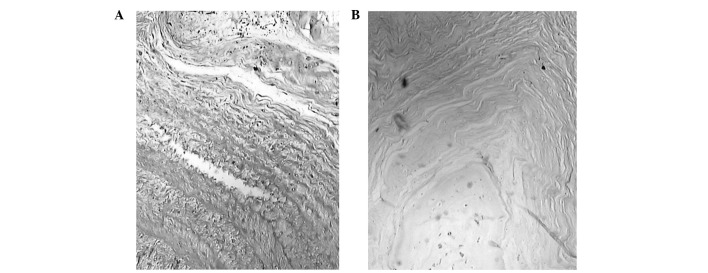
Histological examination using hematoxylin and eosin. (A) Note the marginally interrupted border between the annulus fibrosus and nucleus pulposus, and the tortuosity of the annulus fibrosus. (B) Note the moderate rupture between the annulus fibrosus and nucleus pulposus (magnification, ×100).

At 12 weeks post-cement injection, a slightly interrupted border between the annulus fibrosus and nucleus pulposus, as well as chipping in the nucleus pulposus due to the condensed matrix, were identified in all histological analyses. (Fig. 5). The nucleus pulposus of specific cells was within the adjacent inner annulus fibrosus. The number of cells and cell vacuoles of the nucleus pulposus decreased. Extracellular matrix condensation occurred in certain samples. The cartilage endplate had ruptured or formed serpentine-patterned fibers; however, no calcification occurred in the cartilage endplate. The thickness of the vascular bed was not reduced. Fibrosis was evident in the annulus fibrosus and nucleus pulposus of specific samples from the PMMA groups.

**Figure 6:**
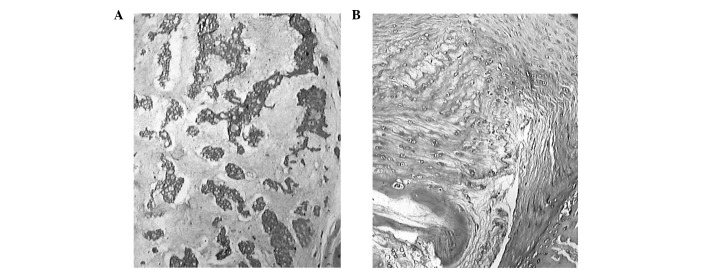
Histological examination using hematoxylin and eosin. (A) The number of cells in the nucleus pulposus was reduced and the extracellular matrix was condensed within the adjacent polymethylmethacrylate. (B) The number of cells in the nucleus pulposus was reduced and the extracellular matrix was condensed slightly within the adjacent calcium phosphate cement (magnification, ×100).

At 24 weeks post-cement injection, no boundary was observed between the annulus fibrosus and the nucleus pulposus. Cells from the nucleus pulposus were observed in the adjacent inner annulus fibrosus, but its vacuoles had shrunk and the number of cells significantly decreased. Disparity in the condensation of the extracellular matrix was observed (Fig. 6). The cartilage endplate had ruptures or serpentine-patterned fibers covering up to 30% of the annulus fibrosus. Various extents of fibrosis were observed in the annulus fibrosus and nucleus pulposus in all samples. Calcification in the cartilage endplate and reduction in the vascular bed were not found in the CPC groups, whereas a thin area of calcification in the cartilage endplate was found in the PMMA groups. Furthermore, necrosis of nucleus pulposus cells, condensation and degradation of the matrix, and rupture and tumescence were more severe in the PMMA groups compared with the CPC groups.

**Figure 7:**
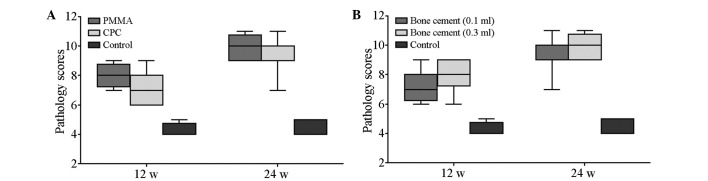
(A) Pathology scores for the PMMA and CPC subgroups in the 12- and 24-week groups. (B) Pathology scores for the various quantities of cement subgroups in the 12- and 24-week groups. PMMA, polymethylmethacrylate; CPC, calcium phosphate cement.

Table III lists the pathology scores of all groups at the two time points. No significant difference was observed in the scores of the control groups at 12 and 24 weeks (t=0.509; P>0.05). The scores of the cement subgroups at 12 and 24 weeks were significantly different compared with those of the control group (P<0.05). The scores of the PMMA group and the CPC group were also significantly different at 12 and 24 weeks. Inter-group variability was accounted for by mixed model analysis (F, 91.52, 13.71, 13.71; P<0.05; Fig. 7).

## Discussion

The most frequently used cement types in PVP are the acrylic polymers, PMMA and CPC. CPC, which is a mixture of several types of calcium phosphate, has osteoconduction and reabsorption properties (13). PMMA is composed of methylmethacrylate polymer and the monomer of PMMA eventually has a toxic effect on cells. PMMA reaches peak temperatures of up to 113°C *in vitro* and produces an exothermic reaction during polymerization (14,15).

During PVP, PMMA may cause thermal necrosis in the adjacent tissue. In particular, direct contact between PMMA and the nerve roots or the dural sac causes thermal necrosis in neural structures. The occurrence and severity of thermal tissue damage depends on the following factors: i) The magnitude and the duration of exposure; ii) the type of tissue exposed; and iii) the extent of local heat convection via blood flow. In the present *in vivo* study, temperatures in the vertebral body and epidural space were not high enough to cause tissue necrosis when only a small quantity (0.5–0.7 ml) of PMMA was injected (16).

Following PMMA injection in the present study, histological examination revealed necrosis of nucleus pulposus cells, condensation and degradation of the matrix, rupture and tumescence of the annulus fibrosus, and calcification of the cartilage endplate. These changes were less prominent in the CPC groups relative to the PMMA groups. Calcification was not detected in the cartilage endplate in the CPC groups. The difference in histological changes between the PMMA and CPC groups may be associated with cell toxicity and the exothermic effect of PMMA.

Affluent veins are present in the vertebral body. Venous flow can efficiently reduce temperature levels and the local concentration of PMMA monomers, preventing structural injury. However, the intervertebral disc has an avascular architecture (15). Following cement leakage, heat convection and toxicity inside the disc cannot dissipate rapidly due to cell infiltration. In the samples injected with PMMA in this study, a calcification zone formed at the cartilage endplate. The cartilage endplate is the main route through which the disc gains nutrition. Thus, calcification accelerates the process of disc degeneration.

The present experiments demonstrate that the degree of disc degeneration positively correlates with the quantity of bone cement injected into each disc. The intervertebral disc is known as a closed buffer system. When cement is injected, the stress in the disc increases as the pressure increases. Intervertebral discs primarily gain nutrition via passive diffusion. The increasing stress may cause cell infiltration hypofunction, which influences nutrient absorption. Furthermore, the metabolic level of the disc cell decreases under sustained high stress. As the level of aerobic oxidation decreases, the lactic acid content increases, and the pH value decreases (17). These changes promote cell death in the disc. Rannou *et al* (18) demonstrated that cell apoptosis in the annulus fibrosus occurred when a mechanical load of 1.3 MPa was applied to the spine of mice. The application of mechanical load increased stress to the disc, which in turn resulted in severe disc degeneration. Another *in vitro* study indicated that a cement-filled bone is 36-fold stronger than a cancellous spinal bone (19).

Injected cement occupies certain spaces in the disc and causes mechanical changes that compress the nucleus pulposus. As a result, the nucleus pulposus is displaced, deformed and broken. The process of disc degeneration is caused by derangement of cell metabolism and dysfunction of biochemical constituents.

Another reason for the degeneration of the nucleus pulposus in the PMMA groups is cell apoptosis. Our histological findings demonstrate that condensation of the extracellular matrix may be caused by several factors: i) Hydratability subsides with an increase in stress, which may be an important factor affecting the extracellular matrix; ii) condensation and degradation of the extracellular matrix are induced by the thermal reaction and toxicity of the PMMA monomer, which may also induce apoptosis; and iii) the quantity and quality of synthesis of the extracellular matrix is reduced with the loss of healthy nucleus pulposus cells. These factors contribute to the degeneration of a cement-injected disc (20).

The present study also indicates that the extent of disc degeneration is affected by the time period following bone cement injection. As the observation time was extended, signals from the nucleus pulposus in T2-weighted images decreased, and the area of relatively high signal declined, accompanied by the irregular appearance of the nucleus pulposus. Histological data reveal the change from a slightly interrupted border at 12 weeks to the loss of any boundary between the annulus fibrosus and nucleus pulposus at 24 weeks. Other key findings include the gradual reduction of the nucleus pulposus cell, the increased significance in the occurrence of matrix condensation, and the extended degree of the ruptured or serpentine-patterned fibers in the annulus fibrosus. These observations, along with the MRI results, strongly demonstrate that degeneration of injected discs increases over time.

Cement leakage, which has a potential influence on the disc during PVP, often occurs under two conditions: Fractured endplates and vacuum clefts (1,21). The most common reason for cement leakage is the fracturing of endplates, which may already exist along with compressive vertebral fractures. Cement leakage may also occur as a result of excessive penetration by the needle into the endplate. Vacuum clefts are caused by a non-union of vertebral fractures. If penetration into the disc space occurs, the risk of cement leakage increases.

The present study used a direct puncture technique and employed injection to disc as the model of cement leakage in PVP. An ideal model for cement leakage should involve injecting cement into the vertebral body through the endplate; however, this model is difficult to manipulate as improper management of the needle tip could injure the endplate. With the trans-vertebral method, controlling the amount of cement leakage may be impossible. Direct puncture with a needle may result in IDD. The degree of IDD has a close correlation with the diameter of the puncture needle, where the smaller the needle, the lower the extent of IDD (12). By using a 27G needle to puncture the disc of a rabbit, An *et al* (22) demonstrated that no histological abnormality occurs in the annulus fibrosus, nucleus pulposus and endplate. Similarly, in the present study, IDD was not observed after using an 18G needle to puncture the discs of dogs in the control group. The reason for the absence of IDD may be associated to the size of the animal relative to the needle.

Although the model used in the present study may not exactly replicate cement leakage during PVP, the data provide evidence of the consequences of leakage. Therefore, caution should be exercised to avoid cement extravasation into the disc space during injection. Prior to cement injection, the operator should verify that the needle tip is outside the disc by using various positions of the C arm when the needle tip is located near the fractured endplate. Cement injection with paste or drainage is recommended in the treatment of vertebrae with vacuum clefts.

## Figures and Tables

**Table I:** Definition of histological grading scale.

Grade	Annulus fibrosus	Border between the annulus fibrosus and nucleus pulposus	Cellularity of the nucleus pulposus	Matrix of the nucleus pulposus
1	Normal pattern of fibrocartilage lamellae (U-shaped in the posterior aspect and slightly convex in the anterior aspect), without ruptured fibers or a serpentine appearance anywhere within the annulus	Normal	Normal cellularity with large vacuoles in the gelatinous structure of the matrix	Normal gelatinous appearance
2	Ruptured or serpentine-patterned fibers in <30% of the annulus	Minimally interrupted	Slight decrease in the number of cells and fewer vacuoles	Slight condensation of the extracellular matrix
3	Ruptured or serpentine-patterned fibers in >30% of the annulus	Moderate/severe interruption	Moderate/severe decrease (≥50%) in the number of cells and no vacuoles	Moderate/severe condensation of the extracellular matrix

**Table II:** MRI indexes (mm^2^ x GY).

Group	12 weeks	24 weeks
Control group	15104.60±3312.17	15762.88±2079.45
PMMA (0.1 ml)	7588.55±1069.59	3875.71±399.61
PMMA (0.3 ml)	5942.18±839.27	1985.58±593.49
CPC (0.1 ml)	10303.23±963.87	5056.15±800.48
CPC (0.3 ml)	9010.63±886.36	3521.01±678.29

Data are presented as mean ± standard error. PMMA, polymethylmethacrylate; CPC, calcium phosphate cement; MRI, magnetic resonance imaging.

**Table III:** Pathology scores in all groups (mean ± SEM).

Group	12 weeks	24 weeks
Control group	4.25±0.46	4.38±0.52
PMMA (0.1 ml)	7.75±0.71	9.63±0.74
PMMA (0.3 ml)	8.25±0.71	10.25±0.71
CPC (0.1 ml)	6.75±0.71	8.75±1.04
CPC (0.3 ml)	7.75±1.04	9.63±0.74

Data are presented as mean ± standard error. PMMA, polymethylmethacrylate; CPC, calcium phosphate cement.
